# Cosmetic, Biomedical and Pharmaceutical Applications of Fish Gelatin/Hydrolysates

**DOI:** 10.3390/md19030145

**Published:** 2021-03-08

**Authors:** Suhair Al-Nimry, Alaa Abu Dayah, Inas Hasan, Rawand Daghmash

**Affiliations:** Department of Pharmaceutical Technology, Jordan University of Science and Technology, P.O. Box 3030, Irbid 22110, Jordan; aaabudayah19@ph.just.edu.jo (A.A.D.); iihasan19@ph.just.edu.jo (I.H.); rmdaghmash19@ph.just.edu.jo (R.D.)

**Keywords:** fish gelatin/hydrolysate, sources, extraction, properties, cosmetic applications, pharmaceutical applications, biomedical applications

## Abstract

There are several reviews that separately cover different aspects of fish gelatin including its preparation, characteristics, modifications, and applications. Its packaging application in food industry is extensively covered but other applications are not covered or covered alongside with those of collagen. This review is comprehensive, specific to fish gelatin/hydrolysate and cites recent research. It covers cosmetic applications, intrinsic activities, and biomedical applications in wound dressing and wound healing, gene therapy, tissue engineering, implants, and bone substitutes. It also covers its pharmaceutical applications including manufacturing of capsules, coating of microparticles/oils, coating of tablets, stabilization of emulsions and drug delivery (microspheres, nanospheres, scaffolds, microneedles, and hydrogels). The main outcomes are that fish gelatin is immunologically safe, protects from the possibility of transmission of bovine spongiform encephalopathy and foot and mouth diseases, has an economic and environmental benefits, and may be suitable for those that practice religious-based food restrictions, i.e., people of Muslim, Jewish and Hindu faiths. It has unique rheological properties, making it more suitable for certain applications than mammalian gelatins. It can be easily modified to enhance its mechanical properties. However, extensive research is still needed to characterize gelatin hydrolysates, elucidate the Structure Activity Relationship (SAR), and formulate them into dosage forms. Additionally, expansion into cosmetic applications and drug delivery is needed.

## 1. Introduction

Gelatin is derived from the parent protein, collagen, which is a ubiquitous fibrous protein found extensively in the tissues of mammalian species. Collagen is the main constituent of connective tissues, bones, cartilages, tendons, skin, and scales [1,2,3,4]. It is made of three strands and is considered a large protein that has a molecular weight around 350 KDa. Each strand has a molecular weight around 105 KDa and is 300 nm long [1,2,4]. Collagen comprises almost all the 20 amino acids. The three chains are connected with each other through hydrogen and covalent bonds, which gives collagen its distinguished level of strength [2,5,6]. Furthermore, the most abundant sequence of amino acids in the triple helix structure is proline (22%), glycine (33%), alanine (11%), hydroxyproline and hydroxylysine. These amino acids are organized in a repeated sequence. There are very slight differences in the components of amino acids of which collagen is constituted according to its source, which results in variable types of collagen (type I, II, III, V, XI, XXIV, XXVII). Type I is the most abundant and comes from the skin, tendons, bones, cornea of the eye, lung, and blood vessels. While type II is derived from cartilages and Type III is the embryonic type. Type IV originates from the basement membranes, and so forth [2,4,5,6,7,8,9,10]. The unique structure of collagen gives it a group of desired characteristics, such as rigidity, flexibility, and strength. These are necessary for its function as the main protein in skin, bones, and tendons [5].

**Figure 1:**
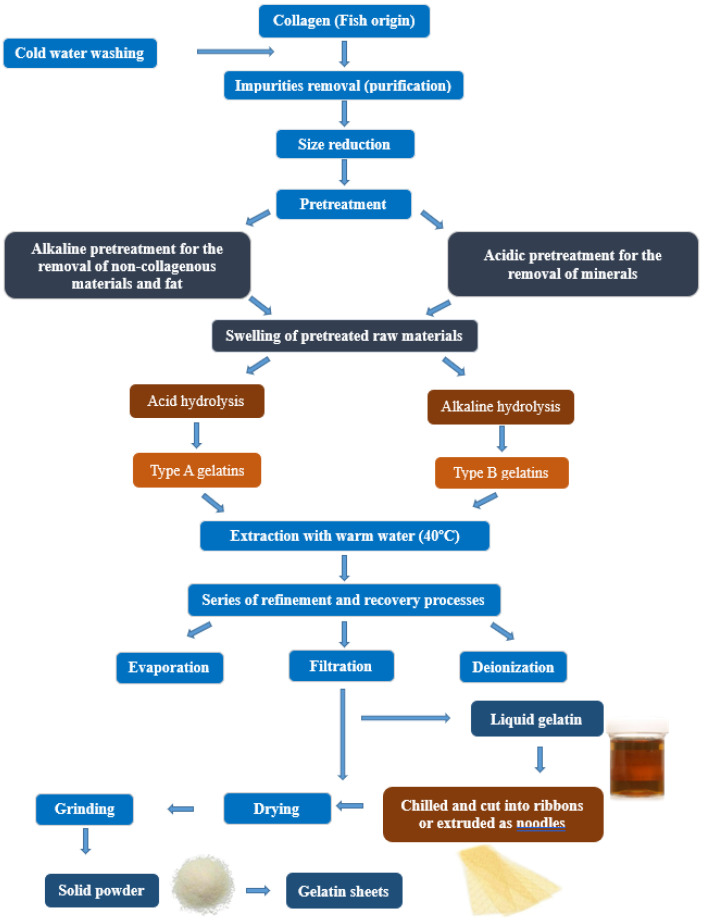
Preparation method of gelatin from collagen.

Gelatin is obtained by partial hydrolysis and incomplete breakage of the crosslinking of collagen molecule [2,11]. The conversion process of collagen to gelatin involves three main steps; the first step involves washing of the source of collagen and pre-treatment with alkali, acid, or enzymes such as pepsin [2,3,12]. The difference in treatments is based on the source of collagen. Acidic treatment is preferable for collagen of the skin of both fish and pigs, which has less crosslinking of covalent bonds, whereas the alkaline treatment is preferable for collagen of the bovine hides that has more covalent bonds and are more complex. This leads to different gelatin types, such as Gelatin A and Gelatin B, which have different features, and different applications [2]. The second step involves heating through water which comprises the actual extraction of gelatin, and the last step includes filtration and drying of gelatin, grinding into solid powder which can be used to prepare sheets [2,7,9,11,12,13,14,15,16]. These different steps are summarized in Figure 1.

Gelatin is derived from many sources and there is an increasing demand on it due to its many applications. The global production of gelatin from porcine skin is considered the highest and accounts for 46% of the overall production. Followed by the production from bovine hides and accounts for 29.4%, and bovine bones and accounts for 23.1% [2]. Gelatin can be derived from various marine species (Fish species, sponges, jellyfish, squid, and snails [17]. Currently, the production from fish accounts for only 1.5 % [2].

Many factors are demanding the discovery of a new source for gelatin production. Firstly, a source that is permissible or lawful to Islamic, Jewish, and Hindus laws: Muslims and Jews are not allowed to consume porcine products, and Hindus are not allowed to consume bovine products. Secondly, safety: gelatin from bovine sources can have the risk of being contaminated with prions that can transmit Bovine Spongiform Encephalopathy (BSE) and foot and mouth diseases [1,2,9,11,18]. Thirdly, environmental preservation and economic revenue: the fishing industry has many by-products that are undesired to consumers such as skin, bones, fins, heads, and scales, which reduces the efficiency and revenue of fish trade. Also, these by-products might end up on shores and cause environmental pollution [3,18,19,20]. Hence, the skin, bones, fins, heads, and scales of the fish can be used as a valuable source of gelatin [19,21].

Comparability of fish gelatin to both porcine and bovine gelatin and comparability of gelatin obtained from different types of fish has been studied. A higher variability of amino acids of collagen obtained from fish than that obtained from beef or pork was found. This higher variability of amino acids of colloagen resulted in higher variability of amino acids of gelatin. The source and type of collagen would affect the properties of the resulting gelatins. Fish collagen/gelatin had lower amounts of proline and hydroxyproline and higher amounts of threonine and serine [9,22,23]. In addition, some studies compared the amino acid content between warm-water fish gelatin (tilapia and Nile perch) and cold-water fish gelatin (codfish). The difference in amino acid content led to variation in the physical and chemical properties of gelatin, and hence to more diverse applications. Cold-water fish gelatin had lower hydroxyproline content. This resulted in lower gel modulus (low gel strength), thermal shrinkage, lower denaturation temperatue and melting (gelling) temperatures (below 8–10 °C) [22,24]. Low melting temperatures allow the use of cold-water fish gelatin in microencapsulation of vitamins, colorants and flavouring agents, like lemon, garlic, black pepper and other flavours [2]. Warm-water fish gelatin had a higher melting point (25–27 °C) than cold-water fish gelatin, but still lower than that of mammalian gelatin (32–35 °C). It melted more easily in the mouth, causing a better release of flavours when compared to mammalian gelatin; therefore, it could be used in the dessert industry. Accordingly, products that are made of warm-water fish gelatin need to be stored at low temperatures, while products made of mammalian gelatin can be stored at room temperature [2,5]. Gelatins are thermo-reversible—they liquefy when heated and form a gel when cooled [7].

Edible solid gelatin, being colourless, odourless and tasteless, can be applied in many industries: (1) Food industry, as a biofilm for meat and other food preservation, since it provides a wall with antimicrobial and antioxidant properties [1,5,9,23,25,26,27,28,29]. These biofilms were also modified using other antimicrobial agents or material such as aloe vera gel [28]. In addition, it is used as a stabilizer and texture enhancer for dairy and bakery products, and in some deserts like gummy candies and marshmallows [2,5,30]. Gelatin has low calories and high protein content, therefore, it used in diet and diabetic food [2]. (2) Cosmetics, it is used in hair gels, creams, lotions, shampoos, and many other products [1]. (3) Biomedical applications, it has many intrinsic activities including antihypertensive, antidiabetic, anticancer, antioxidant, and antimicrobial activities. It is also used in wound dressing and wound healing, gene therapy and tissue engineering [31,32,33,34,35,36,37,38,39,40,41,42,43,44,45,46,47,48,49,50,51]. (4) Pharmaceutical industry and drug delivery, it is widely used as a gelling agent for plasma expanders. In addition, it is used in the manufacture of soft and hard gelatin capsule shells, formulation of tablets, coating of tablets and capsules, microencapsulation of oils and drugs, stabilization of emulsions [52,53], slow-release matrices, medicated sponges, scaffoldings, gels and creams, wound care products, and vaccines [1,2,5,19]. (5) Photographic industry, as a protective film that increases the life span of photos [21,26]. (6) Other industries, like fertilizers and paints [5]. 

In order for fish gelatin to be used in industry, it must be tested for many required specifications such as amino acid composition, white degrees (which indicates the colour of the gelatin (clear and colourless)), gel strength, isoelectric point, ash content, water content, fat content, protein levels, and heavy metals content [26].

This review discusses the cosmetic, biomedical, pharmaceutical and drug delivery applications of fish gelatin/hydrolysate.

## 2. Cosmetic Applications

Gelatin is applied as a gelling ingredient in in many cosmetic products including face creams, body lotions, shampoos, hair sprays, sunscreens and bath salts and bubbles [1].

The skin acts mainly as a barrier against external environmental conditions including exposure to sunlight. Skin ageing is an oxidative process that results in the production of free radicals. It involves intrinsic factors (age), and extrinsic factors (exposure to ultraviolet radiation (A and B)). However, the skin has its own antioxidant defense mechanism. The natural pigment in the skin (melanin) absorbs ultraviolet (UV) light [54]. Fish gelatin hydrolysates (proteins and peptides) were used for the prevention of the damage caused by UV radiation on the skin. They corrected the harm caused to the structure of the skin by maintaining balanced lipids of the skin due to their antioxidant properties [55,56] The exposure to UV radiation leads to reduction of the antioxidant enzymes like total superoxide dismutase (T-SOD), catalase (CAT), and glutathione peroxidase (GSH-Px), which constitute an endogenous system that protects the skin from oxidative stress. Polypeptides derived from Pacific cod (*Gadus macrocephalus*), were studied on mice to determine their counter effect against photoaging and oxidative damage on the skin. The study showed that using gelatin hydrolysates significantly increased the activity of T-SOD, CAT, and GSH-Px. Also, lipid peroxidation was reduced, and down-regulation of inflammatory cytokines levels were seen on experimental mice, which may be due to the inhibition of NF-κB expression by hydrolysed gelatin [56]. In another study, the protective effects of gelatin and its hydrolysates obtained from salmon skin were evaluated on mice skin. Again, gelatin and its hydrolysate were found to lessen the harmful effects of UV radiation by increasing the levels of T-SOD, CAT, and GSH-Px. Another mechanism of gelatin and its hydrolysates in the protection against UV radiation is boosting the immunity by elevating the thymus index and boosting the immune increasing hydroxyproline in the skin, which is an indicator for the collagen content [56]. Similarly, gelatin hydrolysates obtained from fish gelatin of tilapia (*Oreochromis niloticus)* were found to have a scavenging effect against reactive oxygen species of UV that causes damage to the skin and results in early aging [57]. Thus, fish gelatin/hydrolysates could be considered as a novel source of component that have future potential in skin anti-aging products.

## 3. Biomedical Applications

### 3.1. Intrinsic Activities of Fish Gelatin Hydrolystaes

#### 3.1.1. Antihypertensive Activity

Angiotensin-Converting Enzyme (ACE) inhibitors are one of the treatments for hypertension. Attempts to synthesize new ACE inhibitors have produced drug candidates with various side effects, such as dizziness, headache, dysgeusia, cough and rash. Biologically active peptides, such as those in fish gelatin (mainly derived from skin and fish scales), are reasonably priced, abundant and safe, and could be used as antihypertensive ingredients in functional foods to help treat people with high blood pressure [42]. Antihypertensive peptides from fish gelatin exerted their action, by either competitive inhibition or non-competitive inhibition. The presence of C-terminal tryptophan, tyrosine, phenylalanine, or proline, and N-terminal branched-chain aliphatic amino acids is usually an indication of the competitive inhibitory mechanism of peptides. These structural components competed with angiotensin-I to bind ACE and thus prevented the generation of angiotensin-II. The noncompetitive inhibitory peptides formed an inactive complex upon binding to ACE, thus prevented its binding to another substrate. ACE inhibitory peptides with non-competitive mechanism of action, usually had high hydrophobicity at the N-terminus. In general, short-chain peptides with hydrophobic or positively charged residues at the C-terminus were found in the most effective ACE inhibitory peptides. Antihypertensive peptides were extracted from fish gelatin by enzyme digestion, mainly with alcalase, pepsin, trypsin, pronase E and collagenase [42]. Pepsin favored the cleavage between hydrophobic residues, whereas, Arg and Lys- cleavage generally occurred with trypsin hydrolysis [32]. Studies have reported that gelatin hydrolysates from skate skin [36], sea cucumber [37], jellyfish [38], and squid skin [44] had the ability to reduce blood pressure. The findings of these studies are summarized in Table 1.

#### 3.1.2. Antioxidant Activity

Natural antioxidants are attracting more and more attention due to their safety and availability. Peptides derived from collagen could have different levels of antioxidant activity. The peptides that were extracted from fish gelatin hydrolyzed protein had free radicals scavenging activity, inhibited lipid peroxidation, protected DNA from breakage caused by hydroxyl radicals and acted as chelating agents. The antioxidant activities of isolated peptides from different fish derived gelatin are summarized in Table 2.

Although the exact mechanism by which these peptides exhibited the observed antioxidant activity was unknown [42], the following appeared to play a major role in the antioxidant activity:

- The polypeptide molecular weight: lower molecular weight peptides had higher antioxidant activity.
- The presence of hydrophobic amino acids, preferably aromatic amino acids, at the carboxyl and amino terminals.
- Specific free amino acids or sequences, like the dipeptide of Gly-Tyr and the amino acid Tyr, subfractions with peptides rich in Arg, Tyr, and Phe; Tyr.
- Degree of hydrosylation [42,43].

#### 3.1.3. Dipeptidyl Peptidase Inhibitory Activity

Gelatin extracted from salmon trimmings (*Salmo salar*) was hydrolyzed with a variety of proteolytic enzyme preparations. The Dipeptidyl Peptidase (DPP)-IV activity was assessed in vitro for the enzyme hydrolysates, and was found to be the most potent from previously reported values (IC_50_ values ranging from 0.08 to 0.18 mg/mL as opposed to the previously reported >5 mg/mL) [47]. The difference was attributed to the starting materials and the specificity of the enzymes in releasing the potent DPP-IV inhibitory activity [33]. The DPP-IV activity was found to be maintained following enzymatic treatment similar to what is experienced in gastrointestinal digestion, allowing for oral delivery of the hydrolysates [27,33]. When SG-C1 was fractioned using semi-preparative RP-HPLC, four peptides and two free amino acids were identified in a fraction with potent ACE and DPP-IV inhibitory and antioxidant activity. It was determined that the peptide with the Gly-Pro-Val-Ala possess the most potent DPP-IV activity [33]. Further in vivo assessment is needed to assess if these fish gelatin hydrolysates may aid in DPP-IV control in diabetic patients.

#### 3.1.4. Anti-Cancer Activity

The research on the anticancer activities of gelatin and its hydrolysates is limited due to the scarcity of the compounds, and challenges in their extraction, purification and characterization. The current research is limited to in vitro assessment and in vivo studies are necessary to assure the possibility of the use of fish gelatin hydrolysates as cancer treatment [42]. Previous research determined that squid gelatin, as such, did not have any cytotoxic or antiproliferative effect on the studied cell lines (MCF-7 (i.e., human breast carcinoma) and U87 (i.e., glioma)). When squid (*Dosidicus gigas*) gelatin was hydrolyzed with several proteases, the effect of each enzyme hydrosylate on the cell viability of the selected cell line was determined. 1 mg/mL of the hydrolysate was added on the culture medium and changes were recorded after 24, 48 and 72 h [48]. The Esperase hydrolysate had the highest (*p* ≤ 0.05) cytotoxic effect on both cancer cells (96.6 ± 0.5 and 91.2 ± 2.7% of viability inhibition on MCF-7 and U87 cells, respectively, after 72 h). Alcalase hydrolysate also showed relatively high cytotoxic activity (67.8 ± 5.6 and 83.9 ± 4.8% viability inhibition on both cell lines, respectively) [48]. The IC_50_ values for Esperase hydrolysate were 0.13 and 0.10 mg/mL against MCF-7 (i.e., human breast carcinoma) and U87 (i.e., glioma) cell lines, respectively. The IC_50_ values for Alcalase hydrolysate were 0.81 and 0.85 mg/mL against MCF-7 (i.e., human breast carcinoma) and U87 (i.e., glioma) cell lines, respectively. Therefore, squid gelatin hydrolysate may have strong cytotoxic activity against certain types of cancer (such as human breast cancer and glioma) [48].

#### 3.1.5. Antimicrobial Activity

In order for peptides to exert antibacterial effects, they should have less than 50 amino acids, of which nearly 50% are hydrophobic and have a molecular weight of less than 10 kDa. A variety of organisms produce Antimicrobial Peptides (AMPs) as the main innate immune strategy. AMPs played a key role in natural immunity by directly interacting with bacteria and killing it. Compared with conventional bactericidal antibiotics, they killed bacteria faster and were not affected by antibiotic resistance mechanisms [49]. AMPs in fish were cationic peptides with an excess of positively charged Lys, Arg and His residues. The positively charged amino acids of these peptides interacted with the negatively charged molecules in the pathogen membrane and formed pores. The pores in the membrane resulted in the destruction of the bacteria. There were other mechanisms, such as an inhibiting bacterial cell wall, bacterial protein or nucleic acid synthesis, or stimulating autolysate system to lyse bacteria [42,43,49]. AMPs obtained by enzymatic hydrolysis (Alcalase) from tuna and squid gelatin had both antioxidant and antimicrobial activities. These activities increased with decreasing the molecular weight of the fraction [58].

#### 3.1.6. Nutritional Supplement

It has been hypothesized that the nutrition quality of fish gelatin is inadequate on its own, but it is an ideal ingredient for incorporation into nutritional supplements [51]. The amino acid score had been employed to predict protein quality on the basis that the human body needs to get 8 essential amino acids from food to synthesize the body protein. The absence of one amino acid means that the other one cannot be utilized. The essential amino acid index of fish gelatin was below 70, due to the lack of the essential amino acid tyrosine and non-essential amino acid tryptophan and cysteine [42]. However, this theory has been challenged since some proteins did not require 8 essential amino acids for their synthesis [42]. Moreover, the numerous amino acids contained in fish gelatin could be fully utilized by nutritional supplementation [51]. Fish gelatin could thus meet the “ideal protein” requirements by adding the missing essential amino acids directly or indirectly. Given that fish gelatin contain considerable amino acids, obtained from the abundant source of fish skin and fish scales, it may help relieve the shortage of food and nutrition and serve reduce world malnutrition [51]. Additionally, the availability of functional foods to increase Bone Mass Density (BMD) could provide benefits for osteoporosis patients [42].

### 3.2. Wound Dressing and Wound Healing

The use of fish gelatin in wound healing was investigated. Gelatin was extracted from fish processing waste by extrusion-pretreatment and hot water extraction to increase the yield of gelatin. The extracted gelatins (Fish Scale (FS) 2, FS12, and FS14) enhanced cell adhesion, cell growth, and wound healing in HaCaT cells and protected HaCaT cells from H_2_O_2_-induced cellular damage. The highest improvement of cell adhesion, cell growth, and wound healing in HaCaT cells was observed for the FS12 fraction. However, all of the studied gelatins (FS2, FS12, and FS14) aided as natural and effective agents in cell therapies for treating cutaneous wounds [59]. In another study, injectable gelatin microcryogels aided in the management of deep or chronic wounds by transporting cells to the deep layer of the wound tissues [60]. Additionally, oral administration of Chum salmon extracted gelatin enriched the wound healing process in a diabetic rat modal [61].

In order to protect wounds from infection it is inherent that a physical barrier is placed on the wound surface. The optimum wound dressing should retain moisture, absorb extra secretions and be comfortably detached. Important candidates for wound dressing included hydrogels and scaffolds. Hydrogels were formed by chemical or physical crosslinking of soluble or hydrophilic polymers. Although hydrogels had a cooling effect on the wounds, they had weak mechanical integrity when swollen, thus hybrid hydrogels had gained lots of attention in this field [41]. Konjac glucomannan hydrogel is an example of a hydrogel with swelling and gel-forming ability, that could not be adequately utilized due to flexibility and water retention limitations [41]. Fish gelatin gels had been utilized in wound dressing due to their biocompatibility, biodegradability and low antigenicity. The widespread use of either konjac or fish gelatin gels alone in the field of wound dressing was limited as a result of the poor mechanical properties of the two single network gels. A hybrid hydrogel of konjac and fish gelatin was produced by alkaline and thermal treatment. The composite gel had great water absorption and thermal stability, all of which would aid in wound healing. The combination of konjac and fish gelatin was found to be promising to overcome the drawbacks and reinforce the gel properties [41].

**Figure 2:**
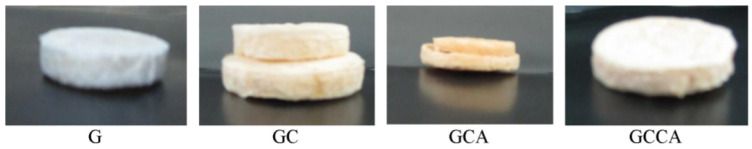
Different types of fish skin gelatin scaffolds. G: simple gelatin; GC: gelatin + chitosan; GCA: gelatin + calcium acetate; GCCA: gelatin + chitosan + calcium acetate [63].

**Figure 3:**
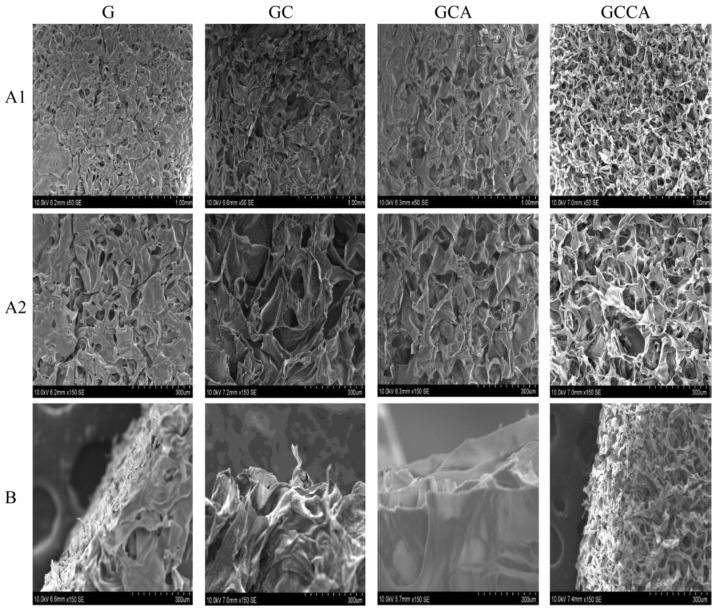
SEM microstructure of different types of gelatin scaffolds. G: simple gelatin; GC: gelatin + chitosan; GCA: gelatin + calcium acetate; GCCA: gelatin + chitosan + calciumacetate. (**A1**,**A2**): surface; (**B**): cross section [63].

Concerning scaffolds, naturally occurring materials have been investigated as alternatives to synthetic materials. As mentioned earlier, the use of fish gelatin is preferred over mammalian sources for environmental and cultural factors; as it is biocompatible and nontoxic to tissues [52]. High protein gelatin derived from biowaste fish scales is a useful therapeutic agent for wound healing applications [62]. The use of bioresorbable Phosphate-based Glass Fibers (PGFs) covered with Fish Scale Gelatin (FSG) extracted from tilapia fish scales for potential wound healing application had been investigated. FSG had a high protein content of 89.4% and was crosslinked with minimal concentrations of glutaraldehyde (GTA) to coat the PGF surfaces. The GTA-crosslinked coated scaffolds were tested in vitro for their cytocompatibility via HaCaT cell adhesion, migration and proliferation studies. The number of adhered cells in Glutaraldehyde-Crosslinked Scaffolds (GCS) 15 samples was significantly more than the uncoated PGFs. GCS10 and GCS15 improved the in vitro artificial wound closure by 27.4% and 28.5% (μm/μm), respectively in wound scratch test, as opposed to the untreated wound. GCS15 resulted in the highest cell viability as well in the Tetrazolium (MTT) assay on day 7. Among all GCS, GCS15 presented the best performance in the tests conducted and could be a promising wound healing material [62]. Lately, the addition of both chitosan and calcium acetate to fish gelatin scaffolds had reduced biodegradation rate in addition to increasing the mechanical features, which was suitable for biomedical applications [63]. These types of fish skin gelatin scaffolds are shown in Figure 2 and their SEM microstructures in Figure 3. Scaffolds is an encouraging technique to permit the fabrication of live functional organs for tissue engineering.

### 3.3. Gene Therapy

As non-viral vectors, gelatin has been adopted in cancer gene therapy [64]. DNA-based components were relocated into cells in gene therapy to eradicate the root causes of diseases rather than alleviate symptoms. Even that porcine gelatin was the most used source of gelatin vector for gene therapy, fish gelatin can also be used due to their comparable properties [65].

### 3.4. Tissue Engineering

Generally, gelatin has benefits over artificial polymers in biomedical uses because of its accessible bioactive motifs in the polymer, high biocompatibility, and little immune response [65,66]. Additionally, gelatin comprises plentiful arginine–glycine–aspartic acid sequences, which encourage cell adhesion, lead to simplify extracellular matrix remodeling [66]. It has low antigenicity, low gelling point, superb solubility but have a low mechanical modulus and undergoes rapid degradation [67]. To overcome these limitations fish gelatin was modified by cross-linking and/or the use of polymers [68]. Crosslinking can be physical, chemical or enzymatic [69]. Several cross-linking agents were used (glutaraldehyde, phenolic acid, 1-Ethyl-3-(3-Dimethylaminopropyl) Carbodiimide (EDC), etc.) [70,71]. FSG was electrospun for 6 hrs to manufacture nanofibrous scaffolds for tissue engineering. The average diameter of 48 ± 12 nm. The nanofibrous scaffolds were physically cross-linked using UV radiation for different time intervals. This was done to improve its water-resistant ability while retaining their biocompatibility. The crosslinking increased the average diameters three folds but did not affect the functional groups of gelatin. A biodegradation study indicated that scaffolds cross-linked for 5 and 10 min. remained in minimum essential medium for 14 days, while scaffolds cross-linked for 20 min. degraded completely after 10 days. All of these scaffolds promoted adhesion and proliferation of human keratinocytes without any toxicity. These scaffolds could serve a wound dressing in the future [72]. Manikandan et al., (2017) modified FSG by crosslinking with phenolic acids and it was found suitable for tissue engineering and regenerative medicine. Following oxidation, a gel-like material, namely, (PAMG)O (oxidized form of phenolic acid modified gelatin), had good mechanical and thermal properties. Additionally, the structure was appreciably porous, which could facilitate the adherence and proliferation of cells [70].

There are many examples on the modification of fish gelatin using polymers [68,73,74,75,76]. For example, cold-water fish skin gelatin was modified with PolyVinyl Alcohol (PVA) and used to prepare a porous three-dimensional sponge for skin tissue engineering. A series of PVA/gelatin scaffolds with different concentrations were prepared and mixed to evaluate the influence of polymer concentration on the structure of the scaffold. The results showed that increasing the polymer concentration affected the dimensions of the pores of the scaffold. The study suggested that PVA/gelatin macroporous scaffolds could be used as a biological matrix for tissue generation [77]. Similarly, fish gelatin methacryloyl hydrogel was prepared using a conventional UV polymerization method after introducing a methacrylamide group to fish gelatin. It was evaluated and compared to porcine methacryloyl hydrogel in terms of physical properties (elastic modulus, degradation and water swelling) and cell behavior (viability, proliferation and spreading). The data suggested that gelatin from cold-water fish may be used to develop engineered biomaterials for drug delivery, regenerative medicine and tissue engineering [67]. Additionally, fish gelatin methacrylate polymer hydrogel mixed with strontium-doped calcium silicate powder was used to fabricate a novel 3D scaffold by photo-crosslinking for bone tissue engineering. This fish-extracted gelatin incorporated with bioactive ceramic enhanced the mechanical property and osteogenic-related behavior of the scaffold [78].

### 3.5. Bone Substitutes

Bone has the ability of self-regeneration due to the complex hierarchical structure, for that it assists as a prototype exemplary in tissue engineering [79]. Fish gelatin sponge was embedded in the femoral condyle of osteochondral defected rabbit, gel foams were biocompatible with little immune response [52]. Moreover, gelatin from fish wastes was combined with bone morphogenetic protein-2 and calcium silicate-based scaffolds to generate an innovative bone morphogenetic protein-2-loaded scaffold [80]. Gelatin sponges in a resorbable gel foam were used as a carrier matrix for human mesenchymal stem cells in cartilage regeneration therapy. The implantation of the gelatin sponge in an osteochondral defect in a rabbit femoral condyle, showed good biocompatibility and no indication of immune response or lymphatic infiltration could be observed at the site [52]. The use of fish gelatin to promote the synthesis of new bone matrix and reduce bone matrix resorption had been applicated in improving osteoporosis. However, current medical therapy did not maintain the beneficial effects after completion of the treatment. The sequence of Asp-Gly-Glu-Ala may have interacted with α2β1 integrin receptors on bone marrow cell membranes to increase the expression of the osteoblast-related gene to promote bone formation. The oral intake of shark skin gelatin Hyp-containing peptides can increase bone formation and the production of type I collagen and proteoglycan in the epiphysis of the bone in ovariectomized rats. The mechanism of synthesis of new bone matrix is not yet fully understood. Fish gelatin hydrolysates was used to limit bone brittleness by increasing the cortical thickness in Mg-deficient mice; it did not affect serum Mg levels. This gave the indication that the mechanism of action is not dependent on Mg and could be due to the abundancy of alanine and glycine in fish gelatin hydrolysates. The expression of type I collagen mRNA and its protein production was notably increased after the intake of gelatin hydrolysates. ALP, a marker of bone formation, was also increased proportionally with gelatin hydrolysates intake. A study concluded that the effect of fish gelatin might be attributed to the reduced expression of the tartrate-resistant acid phosphatase-5b (TRAP-5b) (a marker of bone resorption in serum), and upregulation of the TGF-β1/Smad pathway, an essential signaling pathway in osteoblast collagen synthesis and mineralization. In another attempt to understand the mechanism by which gelatin exerted its effect; synthesized collagen was found to bind and store cytokines and growth factors such as Insulin-like Growth Factor (IGF) I and II and turn on the bone formation process after release from the degraded bone matrix [42]. FS-derived calcium phosphate (CaP) incorporated with gelatin-3-(4-hydroxyphenyl), propionic acid (Gtn- HPA) and carboxymethyl cellulose-tyramine (CMC-Tyr), was used to improve bone formation. The stability of the scaffolds produced by the inclusion of Poloxamer 407 (P407) with cryogel was improved and had a suitable pore size infiltrate cells and promote the regeneration [42].

## 4. Pharmaceutical Applications

### 4.1. Production of Drug Capsules

Capsules are extensively used orally because of their availability, practicality, and ability to cover unkind odors and tastes of drugs [81]. There are two types of the gelatin capsule (i) hard gelatin capsules, generally utilized for powders, and (ii) soft gelatin capsules, generally utilized for liquids. These capsules should be adequately strong and elastic for high-speed filling apparatus and should have softening features for the capsule to seal quickly. The film-forming properties of fish gelatin used in the production of capsules were extensively studied. The main problem was the low gelling temperature because of the low content of proline and hydroxyproline. This problem was overcome by crosslinking with the trans-glutaminase [82]. Examination of fish gelatin thermomechanical properties once added to glycerol and water, indicated that it has comparable performance to porcine and bovine gelatin [83]. In a similar study, the properties of hard capsules made of tuna skin gelatin were studied to determine the ideal gelatin concentration for the manufacture of hard capsules. It was found that 20% gelatin solution was ideal and resulted in capsules with the desired dimensions, weight, pH, disintegration time, and moisture content [84]. Fish gelatin capsules were used for the delivery of algae extract, magnesium peptides, and Betacoten [73]. They were preferred for filling marine supplements such as eicosapentaenoic acid-rich fish oil, spirulina, and cellulose [74].

### 4.2. Coating of Microparticles/Oils by Coacervation/Phase Separation

**Figure 4:**
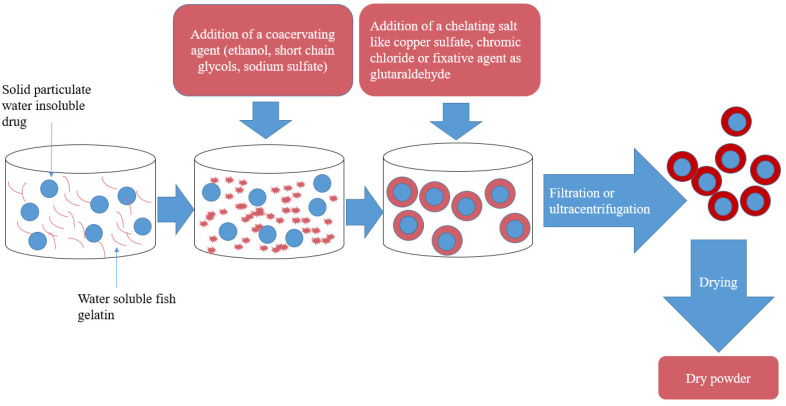
Coating of drug with fish gelatin by coacervation process.

Coating is sometimes needed to mask the bad odor and/or the bad taste of drugs. Multiple methods of coating insoluble particles have been introduced. However, they either lack the ability of good masking of the odor and taste or they suffer from technical problems in processing, such as dealing with the coating material itself. The coating of insoluble particles by coacervation was invented to solve a lot of the problems of the conventional methods [75]. The coacervation process is described briefly as follows: Water immiscible microparticles, like iron oxide and water-soluble polymeric substances, which have film forming ability, such as gelatin, are immersed together in water. After the addition of a coacervating agent like ethanol, short chain glycols or an inorganic salt (sodium sulfate), gelatin is deposited on the minute particles as a hydrophilic wall. This wall is then strengthened and made water insoluble by adding a chelating salt such as copper sulfate, chromic chloride or fixative agent as glutaraldehyde. The resultant minute particles coated with a rigid gelatinous water insoluble material are removed from water by filtration or ultracentrifugation [75,76]. The coacervation process is presented in Figure 4. The coacervation coating method using bovine and porcine gelatin has long been described, but it was not utilized in industry, because both of these gelatin types require to be heated to 40 °C. Most drugs are negatively affected by this temperature. In addition, this amount of heat, makes the process harder to deal with. One main advantage of fish gelatin is its low melting point, and its dissolvability in water at 5–10 °C. This enables coating of heat sensitive and water insoluble drugs with the coacervated fish gelatin coating method. The coating with fish gelatin also provides protection against oxygen and humidity [75]. This process is suitable for almost all drugs except ion exchange resins like cholestyramine, which are resilient to coacervation, possibly due to the charge existing in ion exchange resins. This method produces a range of particles that can be collected using different techniques including filtration, ultracentrifugation, and lyophilization. The particles collected by lyophilization and centrifugation were suspended in water and did not form aggregates [75]. The yield and particle size produced with fish gelatin coacervation coating method is influenced by many factors. These factors include the speed of adding the coacervating substance; the rate by which mixing is done; surfactant addition; duration for the coacervate to form, the conditions at which glutaraldehyde is added, or replaced, and the choice of the collecting process [75]. 

The coacervation method has been applied extensively in the process of microencapsulation of oils. Fish gelatin was used to encapsulate oil droplets of food grade or vitamins like fish oil, which contain omega-3 or lipophilic drugs by the coacervation/phase separation technique. For example, sunflower oil was encapsulated in fish gelatin/gum Arabic complex Coacervate by membrane emulsification. The process started with preparing the oil as emulsion in aqueous media, that contains two polymeric materials; one is a polysaccharide while the other is a protein, like fish gelatin. The aqueous media was maintained at a temperature and pH above the gelling point and the isoelectric point of the used fish gelatin. Phase separation occurred naturally due to the difference in electrostatic charges, where oil droplets were surrounded by the polymeric wall, that was hardened by the addition a crosslinking agent, like Arabic gum [70]. The effect of the total biopolymer concentration, surfactant addition, and concentration of the dispersed phase on the particle size were evaluated. Higher concentration of biopolymers allowed for more charged surfaces and more interaction points to be available; however, more counter ions were released in the solution that decreased the ability of the oppositely charged polypeptides to interact. The addition of anionic surfactant reduced the droplet size of the formed particle because a strong complex was formed between the surfactant and the biopolymer. When the concentration of the dispersed phase in the emulsion was increased from 10 to 30% *v*/*v*, the droplet size remained constant (88 μm); however, when it was increased to over 30% the droplet size increased up to 96 µm [70].

### 4.3. Tablet Coating

Both mammalian gelatin and fish gelatin were used in tablet coating. In addition to gelatin, surfactants (Sodium stearoyl lactylate, calcium stearoyl lactylate and glyceryl mono-stearate), and drying agents (sodium, magnesium or potassium sulfates) were used. The most suitable formula for tablet coating using fish gelatin was [85]:

- Fish gelatin 5–25 *w*/*w* %
- Sodium stearoyl lactylate
- Sodium sulfate
- Propylene glycol monostearate in water
- Sodium acetate
- Sodium lactate.

A plasticizer was added to the formula, to impart softness to the coating especially when the coat was applied directly on the core of the tablet. The preferred plasticizers were propylene glycol monostearate and sodium lactate [85]. The coating was done by dissolving gelatin and other water-soluble ingredients in water, then dispersing the non-water-soluble material in the aqueous solution. Mixing was done on low shear; however, it could be done on high shear, but this was not preferred since foam could form and lead to unequal spraying and other problems. The spraying could be done using nonspecialized equipment like an atomized spray with heated air and fluid bed equipment or a rotating coating pan. Spraying of the tablets was done simultaneously while heat was applied for the coat to dry. Intermittent spraying was not preferred since it required longer cycles [85]. This coating formula could be used for tablets and caplets. It could also be used in coating drugs, vitamins and food supplements and most importantly using conventional equipment. The distinctive factor about this coating formula was that it resulted in a nice, smooth finish, which was appealing to the consumer and facilitated swallowing the tablets. Additionally, it solved many of the outstanding issues with other specialized coating material that required special equipment and techniques like dipping, enrobing, and encapsulating. Moreover, these techniques had high cost with low efficiency. Another advantage of this coating method/formula over others, is that it could be used both directly on the core tablet or on a subcoated tablet, especially when fish gelatin was used.

Subcoating uses a very thin film, usually of hydroxymethyl cellulose, and is colorless or slightly colored to give the desired appearance. Fish gelatin has low bloom strength; thus, gelling did not occur at room temperature. The film formed by gelatin was clear and colorless, however, a colorant or opalescent material could be added to the composition. The weight gain obtained with clear colorless films was between 0.5–1.5% of the tablet weight, while with the colored or opaque films it ranged from 1.5–5.0% of the tablet weight. When overcoating was desired to add gloss to the tablet, the pH needed to be adjusted to give softer coats and prevents cracking. Adjustment could be obtained by the addition of a basic salt into the aqueous solution, like sodium carbonate and sodium tripolyphosphate or dibasic sodium phosphate [85].

### 4.4. Emulsion Stabilizers

Proteins like fish gelatin and polysaccharides like gum Arabic were used as emulsion stabilizers that increase the shelf life and stability of emulsions. Usually, proteins act as emulsifying agents by adsorbing at the oil–water interface. Polysaccharides had a stabilizing role through creating an extended network in the continuous phase, which enhanced the viscosity. The stability of the emulsion was highly influenced by the steric hindrance and electrostatic repulsion between the biopolymers. Destabilization of the emulsion could result from electrostatic attractions. In the study by Avari and Joiner (2017), some of the rheological properties of emulsions, that were formulated with biopolymers either individually or as mixtures were studied. The stability of emulsions was affected by physical and chemical processes that occurred within the components of the emulsion. For instance, the physical instability like creaming was due to changing the spatial arrangement of the components of the emulsion, while chemical instability was due to the changes in the nature of the molecules. Creaming is affected by the pH and for that they have studied the effect of pH on the stability of the emulsion. The studied pH values were pH = 5, pH = 3.6, and pH = 7.0. Fish gelatin has positive zeta potential at pH = 3.6, zero zeta potential at pH = 5.0, and negative zeta potential at pH = 9.0; gum Arabic has negative zeta potential over the entire pH range studied. The hypothesis was that interactions occur between the fish gelatin and gum Arabic in concentrated emulsions at pH = 3.6, and pH = 5, which increased stability. The absence of protein–polysaccharide electrostatic interactions at pH = 9.0 along with the incapacity of the electrostatic repulsive interactions to maintain droplet–droplet separation encouraged creaming [71].

Another study done by Zhang, et al. (2020) compared between the turbidity, gelatin gel strength, water contact angle, Water Holding Capacity (WHC), Fat-Binding Capacity (FBC), emulsifying properties of cold-water fish gelatin and bovine bone gelatin. This was done by determining the Emulsion Activity Index (EAI), Emulsion Stability Index (ESI)), creaming index, and droplet size. Bovine gelatin became turbid at pH 5.0, while fish gelatin remained clear at all pH ranges (Transparency > 97%). Both fish gelatin and bovine gelatin were cooled to a temperature of 10 °C for 16 h. Bovine gelatin formed a gel with a strength of 1050 ± 35 g, while fish gelatin did not form a gel and hence its gel strength and water contact angle could not be determined. However, this was consistent with the fact that fish gelatin has lower gel strength than bovine gelatin. The water contact angle of bovine gelatin was 89 ± 4°. Fish gelatin had lower WHC, while it had higher FHC than bovine gelatin. The WHC for fish gelatin was lower than 100%, but for bovine gelatin, it reached 394 ± 5%. The EAI values of fish and bovine gelatin were increasing with the increase in concentration and decreasing with the decrease in concentration. However, the trend of bovine gelatin was sharper. The size distribution of the droplets of fish gelatin stabilized emulsions was wider than the size distribution of bovine gelatin stabilized emulsions. Creaming indices were similar between both emulsions stabilized by fish gelatin and bovine gelatin. Fish gelatin had lower emulsion stability at 4 °C, because it had greater β-antiparallel and the thinner film structure. Increasing storage temperatures would reduce the differences in stabilization between both fish and bovine gelatin stabilized emulsions [86].

### 4.5. Drug Delivery

The use of natural biomaterials, for example, collagen [68], and gelatin [87], is preferred for drug delivery via different routes because of their biocompatibility compared to synthetic polymers [88]. Significant work has been done using gelatin [87,88]. Gelatin is non-toxic and is biodegradable. Its properties can be further enhanced by modification [89]. For example, modification of gelatin by cross-linking can enhance its stability, and prolong its circulation time in vivo [90]. It can also reduce its expansion in water, lower its porosity to cell membranes, and decrease its solubility at high temperatures [91]. Fish gelatin, particularly that obtained from cold-water fish sources in the non-gelling and non-hydrolyzed form, was utilized as a carrier in a pharmaceutical conformation intended to release the active ingredient quickly [92]. Additionally, fish gelatin was utilized in oral administration to release the active ingredient in the oral cavity after interaction with saliva [51]. The use of fish gelatin in the preparation of microspheres, nanospheres, scaffolds, microneedles, hydrogels, and implants is covered in this part of the review.

#### 4.5.1. Microspheres

Microspheres have enormous surface area and high adsorptive capacity that affects the release profile of drugs. [51]. Additionally, the release profile can be modulated by the modification of the gelatin microspheres by emulsion cross-linking. For example, gelatin (type B) and fish gelatin were used to prepare microspheres loaded with propranolol HCl. Glutaraldehyde was used as a linking agent. The excipients were compatible with the drug. Entrapment efficiency of the optimized batch was 92.38 ± 0.97% and it sustained the release of the drug for more than 11 hours [93]. In another study, fish gelatin microspheres loaded with ciprofloxacin (poorly water-soluble antibacterial agent) were prepared by spray drying. The average diameter of fish gelatin-ciprofloxacin particles was between 2 and 3 μm. The loading efficiency was over 94% and the drug was released within the first 6 h. It was proven that ciprofloxacin had anti-*staphylococcus aureus* and *E. coli* activity and showed minimum inhibitory concentration (MIC) and minimum bactericidal concentration (MBC) values equivalent to pure ciprofloxacin. The spray-drying of the protein-drug particle system was an advantageous alternative to emulsions—it provided higher productivity and had a potential for pulmonary delivery of drugs [94].

#### 4.5.2. Nanospheres

Fish gelatin from tilapia fish skin was used in the production of nanospheres/nanoparticles by using the two-step desolvation method. The first step was the production of low and high molecular weight gelatins. The second step was the precipitation of the nanospheres by the addition of a nonsolvent (acetone) and a cross-linking agent (glutaraldehyde) to the high molecular weight gelatin. The process was optimized by studying the effect of the concentration of acetone, volume of glutaraldehyde and the pH on the size of the nanospheres. The optimized nanospheres (size of 198.46 ± 6.1 nm) were loaded with a model hydrophilic drug (5-fluorouracil) and the release kinetics were evaluated. The release of the drug followed Korsmeyer–Peppas model kinetics (Fickian diffusion). It was concluded that nanospheres made of fish gelatin were a good alternative for the delivery of hydrophilic drugs [95].

#### 4.5.3. Scaffolds

Gelatin-based scaffolds were utilized as a vehicle for delivery of many bioactive materials and drugs, for example, growth factors, Poly(lactic-co-glycolic acid) (PLGA) and curcumin, ciprofloxacin, and dexamethasone [96,97,98,99] The existence of growth factors within extracellular matrix imitating scaffolds is substantial for tissue guidance, repair, and development [100]. Consequently, a spatially and sustained/controlled delivery of growth factors should lead to more effective neo-tissue growth [101]. In a recent study, fish gelatin scaffolds cross-linked with three different materials were tested as substrates for fibroblast growth factors. The scaffolds cross-linked with glutaraldehyde were the most suitable [102]. Additionally, porous scaffolds were beneficial in numerous biological, and biomedical applications [103]. Porous fish gelatin-based scaffolds were fabricated as a carrier for tetrahydrocurcumin, an antibacterial agent [104]. Similarly, an electrospun fish gelatin nanofibrous mat for drug delivery of was fabricated and compared to a conventional fish gelatin film. It was loaded with a model hydrophilic drug, caffeiene. The diameter of the drug-loaded nanofibers was in the range of 200–220 nm. The drug was incorporated into the fish gelatin nanofibers in the amorphus form as compared to the crystalline form in conventional fish gelatin film. The fish gelatin nanofibrous mat had good flexibility compared to the film due to its interporous nanofiber network. The disintegration and the drug release from the nanofibers was much faster than the disinegration and drug release from the conventional film [105].

#### 4.5.4. Microneedles

**Figure 5:**
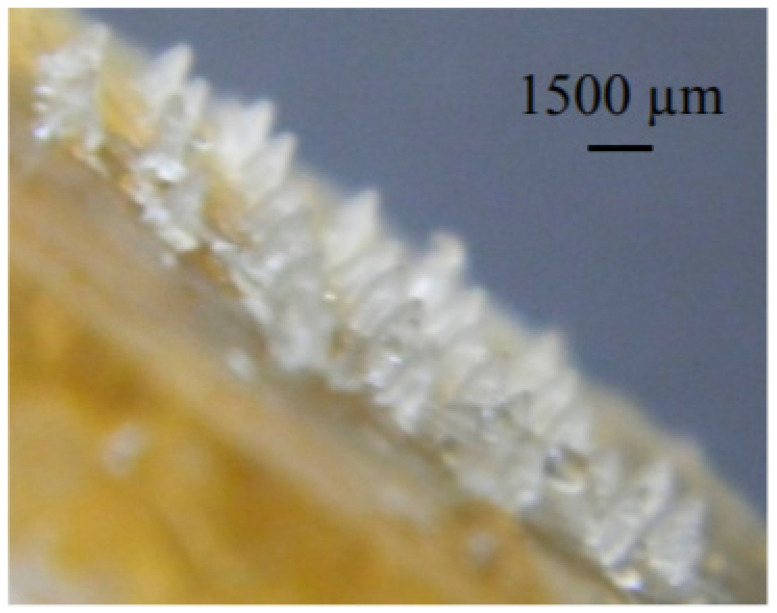
1500 µm-long microneedle array formed from fish scale biopolymer at 50 °C [115].

**Figure 6:**
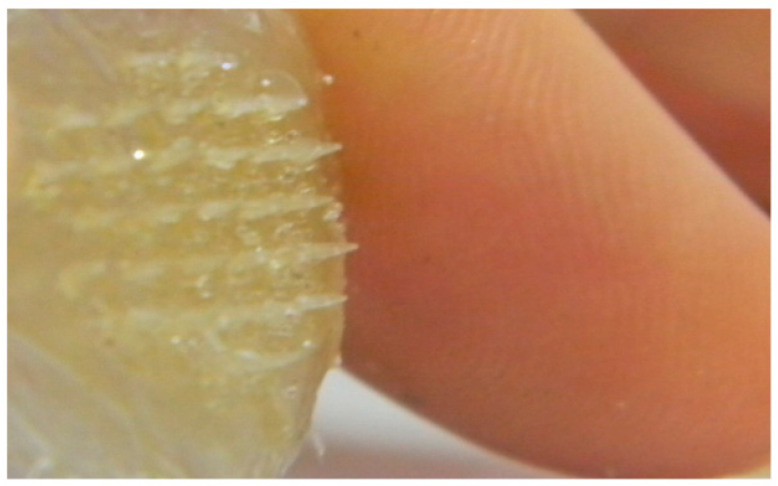
Microneedle array formed from fish scale biopolymer-nanocellulose 20 g at 80 °C placed next to a human finger [115].

The microneedle array is one of the best methods for improving the transdermal entrance of compounds, for example, therapeutics, vaccines, and proteins into the body in a negligibly invasive way. Microneedles were manufactured from many materials, for example, polymers, metals, and silicone [106]. Biocompatible metal microneedles were somewhat costly and required expensive production processes. Alternately, silicone microneedles were generally fragile [107]. On the other hand, polymeric microneedles were reasonable in terms of cost and could utilize reproducible production methods [108]. Recently, scientists focused on the development of non-toxic and biodegradable polymeric microneedles which have strong microstructures to penetrate the skin [109,110]. Natural polymers were favored over synthetic polymers again because of their availability, biodegradable, inexpensive, and non-toxic nature [111]. Microneedles manufactured from biopolymers such as fish gelatin had a capability to penetrate the skin [112]. Also, the delivery of gelatin and collagen themselves or their hydrolysates to the skin had displayed some bioactive properties, e.g., mineral binding capacity, antimicrobial activity, improving skin elasticity, bone strength, immunomodulatory activity, and lipid-lowering effect [113]. Biocompatible microneedles from fish gelatin were prepared and their ability to insert and dissolve into the skin as a drug delivery device were evaluated [114]. In another study, microneedles were synthesized using fish scale biopolymer (FSBP) alone or FSBP and nanocellulose crystals. This was done to enhance the water absorption, water stability and mechanical properties of the microneedle. FTIR studies confirmed that the biopolymer extracted from the FS was gelatin [115]. The FSBP microneedle array is shown in Figure 5, while the FSBP and nanocellulose crystals microneedle array is shown in Figure 6.

#### 4.5.5. Hydrogels

Hydrogels are hydrophilic polymers that are crosslinked through chemical reactions. The polymers could be natural or synthetic. Hydrogels have many biomedical applications, for example, in tissue engineering, drug delivery, and regenerative medicine [116]. According to their high biocompatibility and low immune response, naturally sourced polymers were preferred over synthetic polymers [66]. Fish gelatin has a low viscosity and a low gel point below 10 °C, while mammalian gelatin remains in a gel state and has a high viscosity at room temperature [117]. The gelling behavior of fish gelatin promotes the improvement of systemic injection technology and may increase the release in vivo [118]. Furthermore, fish gelatin has higher emulsion stability than bovine gelatin, so it is appropriate for the creation of emulsion innovation [119]. Besides, fish gelatin utilization is environmentally favorable and cost-effective as the material sources since it is obtained from numerous by-products of fish wastes [120]. As mentioned earlier, a hybrid hydrogel of konjac and fish gelatin was produced by alkaline and thermal treatment. It was loaded with matrine. Matrine is the main active ingredient of many Chinese herbal medicines such as *sophora flavescens*, *sophora alopecuroides*, *radix sophora*, etc. It had been adopted for the treatment of vaginitis, chronic cervicitis, senile vaginitis and pelvic inflammatory disease clinically, since it possesses a variety of pharmacological effects, such as bacterial activity, anti-inflammatory, anti-tumor, etc. The research indicated that Matrine-loaded konjac-fish gelatin hydrogel could preserve an environment helpful to wound healing and had an antimicrobial effect on the wound surface. [41]. Fish gelatin can be modified as mentioned earlier by cross-linking or by combination with polymers. For example, fish gelatin methacryloyl hydrogels were synthesized using conventional UV polymerization methods and compared with porcine gelatin methacryloyl hydrogels in terms of cell behavior and physical properties. The results showed the feasibility of using fish gelatin methacryl hydrogel to replace mammalian gelatin methacryl hydrogel in tissue engineering, regenerative medicine and drug delivery [67]. 

In another study, a double network composite hydrogel based on PVA and fish gelatin was synthesized via thermal treatment and repeated freeze-thawing. The composite hydrogel showed a high ability to absorb fluids. Salicylic acid was added to the hydrogel to exert antibacterial properties; it showed good sustained release properties verified by activity tests. These results demonstrated that PVA-fish gelatin based interpenetrating hydrogel is an appropriate biomaterial for drug-carrying wound dressing application [121]. In a similar study, a 3D-hydrogel patches were printed using fish gelatin and used for the local delivery of PEGylated liposomal doxorubicin. The main component of the printer ink was semi-synthesized fish gelatin methacryloyl (F-GelMA), derived from cold fish gelatin. Carboxymethyl cellulose sodium was added to improve the properties of F-GelMA (low viscosity) by photopolymerization. Drug release was dependent on the shape of the 3D-printed patches and UV-LED exposure time [122]. Hydrogel pads were manufactured using fish gelatin obtained from tilapia fish scales and cross-linked with carboxymethyl cellulose and loaded with Thai herbal plai formula, which is used for treating pain and inflammation. This pad can be used as a medicated pad for patients that are allergic to paper or plastic pads [123]. Another hydrogel manufactured using fish gelatin and crosslinked using glutaraldehyde was used for the transdermal delivery of salicylic acid and 5-sulfosalicylic acid. Drug release followed Fickian diffusion and the diffusion parameters decreased with decreasing the hydrogel pore size and increasing the drug size [124].

**Figure 7:**
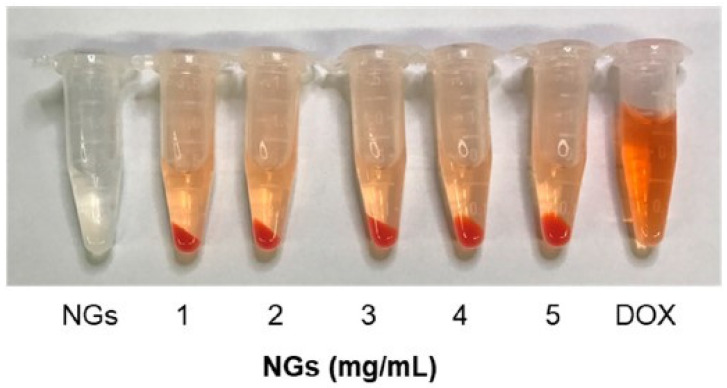
Photos of fish gelatin methacryloyl (GelMA) nanogels, doxorubicin (DOX)-loaded GelMA NGs, and free DOX after centrifugation [128].

**Figure 8:**
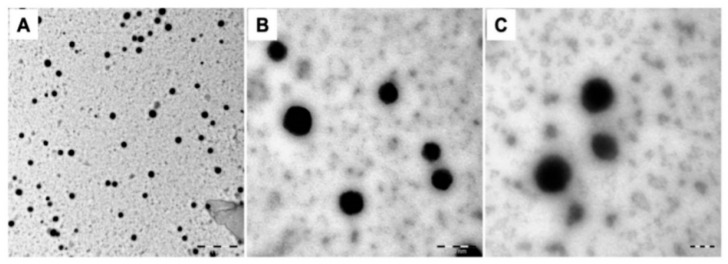
Transmission electron microscopy images of gelatin methacryloyl nanogels (GelMA NGs). (**A**) Scale bar = 1 µm; (**B**) scale bar = 200 nm; (**C**) scale bar = 100 nm [128].

Hydrogels that are crosslinked 3D polymer networks at the nano-scale are termed nanogels [125]. There are many benefits of the nano-sized drug delivery vehicles in systemic uptake; for example, decreasing the side effects of drugs, improving drug stability, and improving retention and permeability outcome [126]. Additionally, the porosity of nanogels increases the drug-loading ability when compared to other solid nanoparticles, such as silica nanoparticles and gold nanoparticles. It also increases the swelling property which facilitates the controlled release. The mechanical stability of nanogels can be enhanced via chemical cross-linking, resulting in a wider application for drug delivery [127]. A recent study examined the formation of nanogels using fish gelatin methacryloyl and the probability of their use in the delivery of doxorubicin [128]. Photos of gelatin methacryloyl nanogels loaded with the drug are shown in Figure 7 and Transmission Electron Microscopy (TEM) images (GelMA NGs) are shown in Figure 8. The release of the drug from the nanogels was pH-dependent and sustained.

## 5. Conclusions

Fish gelatin is extracted from collagen obtained from by-products of the fish industry by simple methods. It is biocompatible, biodegradable, safe and can be used as another source of gelatin in addition to the mammalian sources (bovine and porcine). A source that is permissible or lawful to Islamic, Jewish, and Hindus laws.

Fish gelatin has lower rheological properties and lower melting points than mammalian gelatin. Additionally, cold-water fish gelatin has lower melting point than warm-water fish gelatin. This makes it more suitable for certain applications for example, microencapsulation of oils. On the other hand, it can be easily modified by cross-linking or the use of polymers to enhance its mechanical properties and make it more suitable for other applications, for example, tissue engineering.

Fish gelatin hydrolysates have many intrinsic activities. However, further studies are needed to elucidate the structure of fish gelatin hydrolysates that are active specially as anticancer and antidiabetic agents, characterize them, elucidate the structure activity relationship and deliver them using suitable routes of administration. These studies are needed to reveal new intrinsic activities.

The application of fish gelatin/hydrolysate in cosmetics is mostly restricted to protection against UV radiation. Also, its application in drug delivery is still modest, and expansion in these two fields is needed. For example, the use of films loaded with drug for local delivery to the skin or for systemic drug delivery is a potential application.

## Figures and Tables

**Table 1:** Antihypertensive activity of gelatin hydrolysates obtained by enzymatic treatment from different types of fish.

Fish Part	Enzymes	Isolated Peptides	IC_50_	Reference
Alaska pollack skin	Alcalase, pronase E, and collagenase	LGP, GLP, PLG, LPG and PGL	0.72, 1.62, 4.74, 5.73 and 13.93 mM	[31]
Nile tilapia skin	Bromelain, papain, trypsin, flavourzyme, alcalase and neutrase	Not determined	Antihypertensive activity: 89–93%	[32]
Salmon (skin, bone, and residual meat)	Corolase PP and Alcalase 2.4 L in combination with flavourzyme 500 L	FG-C1 * (4 peptides) (Pro-Pro, Gly-Phe, Gly-Pro-Val-Ala and Gly-Gly-Pro-Ala-Gly-Pro-Ala-Val) and 2 free amino acids (Arg and Tyr)	0.13 and 0.28 mg/mL	[33]
Nile tilapia gelatin (commercially provided)	Alcalase	DPALATEPDPMPF	Antihypertensive activity: 52%	[34]
Tilapia skin gelatin	Simulated GI Digestion	VGLPNSR, QAGLSPVR	80.90, 68.35 μM	[35]
Skate (*O. kenojei*) skin gelatin	Alcalase and alcalase/protease	LGPLGHQ, MVGSAPGVL	4.22 and 3.09 μM	[36]
Sea cucumber (*Acaudina molpadioidea*) body wall	Bromelain and alcalase	Peptide of five main amino acids (Glu, Asp, Pro, Gly and Ala)	0.0142 mg/ml	[37]
Jellyfish (*Rhopilema esculentum*) whole	Alcalase	UF3-B2 * was rich in Gly, Pro, Glu, Ala, and Asp	0.043 mg/ml	[38]

* FG-C1 & UF3-B2: the peptide hydrolysate fractions with the most activity.

**Table 2:** Antioxidant activity of isolated peptides from different fish-derived gelatin.

Fish Part	Enzymes	Isolated Peptides	Assessment of Activity	Reference
Nile tilapia skin	Bromelain, papain, trypsin, flavourzyme, alcalase and neutrase	Not determined	ABTS, lipid peroxidation methods, FRAP, and ferrous ion chelating	[32]
Salmon (skin, bone, and residual meat)	Corolase PP and Alcalase 2.4 L in combination with flavourzyme 500 L	FG-C1 * (4 peptides (Pro-Pro, Gly-Phe, Gly-Pro-Val-Ala and Gly-Gly-Pro-Ala-Gly-Pro-Ala-Val) and 2 free amino acids (Arg and Tyr)	Corolase hydrolysates more potent (ORAC) activities than intact SG	[33]
Hoki skin	Trypsin, R-chymotrypsin, and pepsin	His-Gly-Pro-Leu-Gly-Pro-Leu	Linoleic acid peroxidation and the activity was closer to the highly active synthetic antioxidant butylated hydroxytoluene.	[45]
Amur sturgeon skin	Alcalase or flavourzyme	Oligopeptide; N.D.	Preventing lipid oxidation as evidenced by the lower TBARS formation	[46]

* FG-C1 & UF3-B2: the peptide hydrolysate fractions with the most activity.
